# VISTA: an integrated framework for structural variant discovery

**DOI:** 10.1093/bib/bbae462

**Published:** 2024-09-19

**Authors:** Varuni Sarwal, Seungmo Lee, Jianzhi Yang, Sriram Sankararaman, Mark Chaisson, Eleazar Eskin, Serghei Mangul

**Affiliations:** Department of Computer Science, University of California Los Angeles, 580 Portola Plaza, Los Angeles, CA 90095, United States; Department of Computer Science, University of California Los Angeles, 580 Portola Plaza, Los Angeles, CA 90095, United States; Department of Quantitative and Computational Biology, Dana and David Dornsife College of Letters, Arts and Sciences University of Southern California, 3540 S Figueroa St, Los Angeles, California 90089, United States; Department of Computer Science, University of California Los Angeles, 580 Portola Plaza, Los Angeles, CA 90095, United States; Department of Quantitative and Computational Biology, Dana and David Dornsife College of Letters, Arts and Sciences University of Southern California, 3540 S Figueroa St, Los Angeles, California 90089, United States; Department of Computer Science, University of California Los Angeles, 580 Portola Plaza, Los Angeles, CA 90095, United States; Department of Quantitative and Computational Biology, Dana and David Dornsife College of Letters, Arts and Sciences University of Southern California, 3540 S Figueroa St, Los Angeles, California 90089, United States; Department of Clinical Pharmacy, Alfred E. Mann School of Pharmacy, University of Southern California, 1540 Alcazar Street, Los Angeles, CA 90033, United States

**Keywords:** bioinformatics, computational biology, machine learning, structural variation

## Abstract

Structural variation (SV) refers to insertions, deletions, inversions, and duplications in human genomes. SVs are present in approximately 1.5% of the human genome. Still, this small subset of genetic variation has been implicated in the pathogenesis of psoriasis, Crohn’s disease and other autoimmune disorders, autism spectrum and other neurodevelopmental disorders, and schizophrenia. Since identifying structural variants is an important problem in genetics, several specialized computational techniques have been developed to detect structural variants directly from sequencing data. With advances in whole-genome sequencing (WGS) technologies, a plethora of SV detection methods have been developed. However, dissecting SVs from WGS data remains a challenge, with the majority of SV detection methods prone to a high false-positive rate, and no existing method able to precisely detect a full range of SVs present in a sample. Previous studies have shown that none of the existing SV callers can maintain high accuracy across various SV lengths and genomic coverages. Here, we report an integrated structural variant calling framework, Variant Identification and Structural Variant Analysis (VISTA), that leverages the results of individual callers using a novel and robust filtering and merging algorithm. In contrast to existing consensus-based tools which ignore the length and coverage, VISTA overcomes this limitation by executing various combinations of top-performing callers based on variant length and genomic coverage to generate SV events with high accuracy. We evaluated the performance of VISTA on comprehensive gold-standard datasets across varying organisms and coverage. We benchmarked VISTA using the Genome-in-a-Bottle gold standard SV set, haplotype-resolved de novo assemblies from the Human Pangenome Reference Consortium, along with an in-house polymerase chain reaction (PCR)-validated mouse gold standard set. VISTA maintained the highest F1 score among top consensus-based tools measured using a comprehensive gold standard across both mouse and human genomes. VISTA also has an optimized mode, where the calls can be optimized for precision or recall. VISTA-optimized can attain 100% precision and the highest sensitivity among other variant callers. In conclusion, VISTA represents a significant advancement in structural variant calling, offering a robust and accurate framework that outperforms existing consensus-based tools and sets a new standard for SV detection in genomic research.

## Introduction

Genomic regions with altered deoxyribonucleic acid (DNA) sequences caused by deletion, duplication, insertion inversions, and other complex rearrangements are known as structural variants. Structural variations (SVs) are present in approximately 1.5% of the human genome [1]. Still, this small subset of genetic variation has been implicated in the pathogenesis of psoriasis [2], Crohn’s disease [3] and other autoimmune disorders [4], autism spectrum and other neurodevelopmental disorders [5–8], and schizophrenia [9–12]. Since identifying structural variants is an important problem in genetics, several specialized computational techniques have been developed to detect structural variants directly from sequencing data.

Substantial differences exist in the number of identified variants in SV catalogs published during the past decade. The 1000 Genomes Project SV dataset identified over 68 000 SVs [13]; a genome-wide survey of 769 Dutch individuals identified approximately 1.9 million structural variant [14]; and a survey based on profiled whole genomes of 14 891 individuals across diverse global populations identified 498 257 SVs [15]. In addition, discrepancies in the number of SVs reported by these methods suggest that SV callers may fail to detect SVs and may report false positives (FP; i.e. SVs that do not exist). Since detecting SVs using short-read sequencing is a challenging problem, a variety of different SV detection methods have been developed. Typically, SVs are detected by looking for changes in read depth (e.g. GROM [16]), identifying clusters of discordantly aligned paired-end (PE) reads (BreakDancer [17]) or split reads (e.g. Octopus [18]a), constructing assemblies or a combination of these approaches. Read-pair methods leverage information about deviant pair distances and orientations in PE sequencing data. Read-depth methods are based on the assumption that the sequencing depth over a genomic region is proportional to its copy number. Consequently, deviations from the expected depth may indicate a deletion or duplication. Split-read methods utilize reads that are partially aligned to the reference genome, indicating a potential structural variant at the breakpoint. Assembly-based methods create a de novo assembly of the genome and then align the assembly to a reference genome to detect structural variants. Hybrid methods combine one or more of the above approaches to increase sensitivity and specificity. This diversity of approaches also results in a variation in the performance across SV types and sizes, as well as varied compute requirements. Consensus-based SV callers, such as Parliament2 [19], Jasmine [20], and SURVIVOR [21] callers, perform ensemble optimization by combining the outputs of multiple SV callers into a high-quality consensus set. Although a plethora of SV detection methods have been developed, dissecting SVs from whole-genome sequencing (WGS) data presents several challenges, with the majority of SV detection methods prone to from a high false-positive rate [22], and no existing method able to accurately detect a full range of SV’s present in a sample.

To address these challenges, we developed Variant Identification and Structural Variant Analysis (VISTA), an automated SV detection algorithm integrating multiple variant callers. VISTA automatically executes the combination of individual callers and can achieve an optimal balance between precision and sensitivity. We have extensively evaluated the performance of VISTA using three comprehensive datasets with highly accurate gold standard benchmarking data across 17 WGS samples, namely the Genome-in-a-Bottle (GIAB) gold standard SV set, haplotype-resolved de novo assemblies from the Human Pangenome Reference Consortium [23,24] (HPRC), along with an in-house PCR-validated gold standard across seven mouse strains to demonstrate its superiority, in consistently maintaining the highest F1 score among top consensus-based tool. Notably, HPRC represents a highly accurate SV set consisting of haplotype-resolved de novo assemblies and was never used for a comprehensive assessment of the accuracy of SV callers. Using prepared benchmarks, we compare the performance of VISTA with 21 individual SV callers (Octopus [18], Pindel [25], Manta [26], CLEVER [27], DELLY [28], PopDel [29], BreakDancer [17], GASV [30], Smoove [31], GenomeSTRiP [32], MiStrVar [33], indelMINER [34], GRIDSS [35], Tardis [36], CREST [37], RDXplorer [38], transIndel [39], LUMPY [40], GROM [16]), and three popular consensus-based callers (Parliament2 [19], SURVIVOR [21], and Jasmine [20]) to demonstrate VISTA’s ability to balance precision and sensitivity with the highest F-score, consistently across different organisms, variant length categories, and data of varying genomic coverages. VISTA obtains the highest F-score of 0.785 on human full coverage data, of 0.769 on full coverage mouse data, and of 0.51 on WGS data with coverage less than 0.5×. VISTA represents a transformative step forward in the field of structural variant calling, providing a powerful and accurate framework that surpasses existing consensus-based tools and establishes a new benchmark for SV detection in genomic research.

## Methods

### Preparing the datasets

We utilized publicly available benchmark data from the GIAB consortium, specifically the Ashkenazi Jewish Trio son (NA24385/HG002). The GIAB-HC SV truth set is based on HG002, a male Ashkenazi Jewish sample using multiple technologies and manual vetting of the SV. While VISTA can infer multiple SV types, the current GIAB call set largely comprises insertion and deletion events. Publicly available alignment files on the GIAB website were used as input to variant callers. The average coverage depth was 45× and the reads were 2 × 250 bp PE reads. We used the preliminary variant set from GIAB, containing variants in HG002, as our gold standard. Since variants with lengths less than 50 bp are not characterized as structural variants, we filtered all variants smaller than 50 bp [41]. The set contained 71 152 variants, out of which 8190 deletions remained after extracting variants with lengths over 50 bp, and 4120 deletions remained after filtering the high-confidence regions. Furthermore, 14 779 insertions remained after filtering insertions with lengths greater than 50 bp, and 6723 insertions remained after filtering the high-confidence regions.

In order to demonstrate the scalability of VISTA across a large number of samples and gold standard sets, we tested VISTA on the following 10 randomly selected samples of human data: HG00733, HG00438, HG00621, HG00735, HG00741, HG01071, HG01106, HG01109, HG01243, and HG01175. The alignment files are from the 1000 Genomes Project with 30× coverage and 2 × 150 bp reads on the reference GRCh38 [42]. They are publicly available through the International Genome Sample Resource (IGSR) portal [43]. VISTA and other variant callers take these alignment binary alignment and map (BAM) files as input and output SV sets. We used dipcall [44] to generate SVs of the human samples directly from haplotype-resolved de novo assemblies produced by the HPRC. The HPRC samples are composed of different populations with genetic diversity, including 51% African, 34% American, 13% Asian, and 2% European samples.

We utilized a collection of homozygous deletions found within inbred mouse chromosomes to demonstrate VISTA’s generalizability across organisms. More specifically, we deliberately restricted the deletions to mouse chromosome 19 due to its being the smallest. We used a PCR-validated set of deletions, in which the mouse deletions were manually curated. To precisely determine the ends of each deletion at the base pair, we employed targeted PCR amplification of the breakpoints followed by sequencing. The read alignment file used for the manual curation of deletions was also provided as input to the structural variant callers. This approach makes our gold standard complete, encompassing all possible true deletions or positives. To ensure that our gold standard is complete with respect to the alignment file, we manually examined all possible deletions and subsequently validated each one by PCR. While our gold standard may not be universally complete, it was complete concerning the alignment files provided to the SV callers, as every deletion detectable from the alignment was recorded and further validated by PCR. The set of deletions we used among seven inbred strains, called with reference to C57BL/6 J, is illustrated in Fig. 2a and listed in Supplementary Table 5. We filtered out deletions shorter than 50 bp [41] since structural variants are genomic events with lengths greater than 50 bp. High-coverage sequence data was used as input to the SV callers in the form of aligned reads. Reads were mapped to the mouse genome (GRCm38 Mouse Build) using BWA with - a option. Additional information on the gold standard, raw data preparation, and analysis can be found in the Supplementary materials.

**Table 1:** Datasets used in this study grouped by the number of samples and sample names (indicated in the columns “# Samples” and “WGS Samples”. We documented how each of the gold standard datasets were prepared (“Method of preparation”) and recorded the read length and organism corresponding to the dataset. We also recorded the paper where the dataset was first published (“Citation”).

Dataset name	# Samples	Method of preparation	Organism	WGS samples	Reference	Read length
GIAB high confidence set (GIAB-HC)	1	Integrate multiple short- and linked-read sequencing datasets	Human	HG002	Zook, Justin M., et al. “An open resource for accurately benchmarking small variant and reference calls.” Nature biotechnology 37.5 (2019): 561-566.	2 × 250 bp
Human Pangenome Reference Consortium (HPRC-HC)	10	Haplotype-resolved de novo assemblies from HPRC	Human	HG00733, HG00438, HG00621, HG00735, HG00741, HG01071, HG01106, HG01109, HG01243, HG01175	Wang, Ting, et al. “The Human Pangenome Project: a global resource to map genomic diversity.” Nature 604.7906 (2022): 437-446.	2 × 150 bp
Mouse gold standard set (7MM-HC)	7	In-house PCR validated	Mouse	A_J, AKR_J, BALB_CJ, C3H_HeJ, CBA_J, DBA_2J and LP_J.	Sarwal, Varuni, et al. “A comprehensive benchmarking of WGS-based deletion structural variant callers.” Briefings in Bioinformatics 23.4 (2022): bbac221.	2 × 100 bp

### Variant Identification and Structural Variant Analysis algorithm

**Figure 1:**
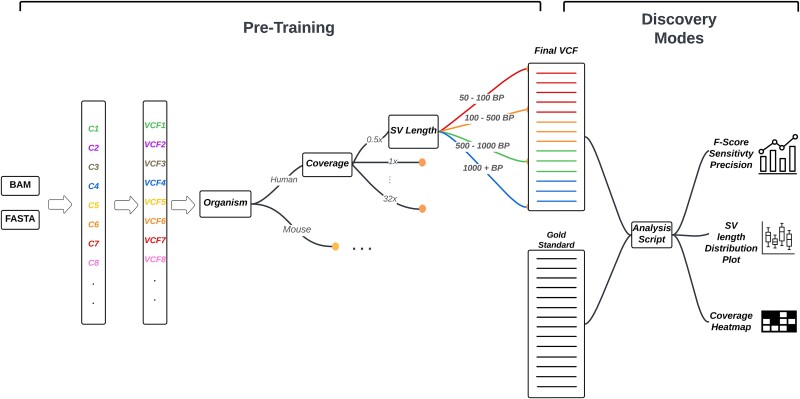
Overview of the approach implemented in VISTA. In the training phase, VISTA inputs BAM and FASTA files and produces VCF files for each caller. VISTA is then trained to determine the optimal combination of callers based on the organism, coverage, and variant length. In the test phase, VISTA uses its pre-trained algorithm to combine the VCF files from individual callers to produce a high-accuracy VCF.

VISTA is a consensus-based variant caller that produces high-accuracy call sets based on the input of individual variant callers using a novel merging and filtering algorithm (Fig. 1). VISTA takes the output of individual callers as inputs and produces a highly accurate file predicting variants, in Variant Call Format (VCF) format. VISTA consists of two states, pre-training and discovery. In the pre-training stage, we use datasets with a well-defined gold standard. First, a list of input VCF files is provided to VISTA as input, containing variants called by individual top-performing callers. Next, VISTA identifies which organism is being called, the genomic coverage of the input sample, as well as the length distribution of the predicted variants. Then, based on the parameters above, namely the organism type, coverage, and length, VISTA bins the input VCFs into different categories. The variant length bins were set to 50–100 bp for short deletions, 100–500 bp and 500–1000 bp for medium-sized deletions, and 1000+ bp for long deletions. These bin sizes were chosen based on a permutation analysis (Supplementary Table 8), and the rationale was to select a diverse set of callers that demonstrate a significant difference in the performance across bins. The comprehensive gold standard is used to evaluate metrics such as the sensitivity, precision, and F-score for each bin. VISTA then uses a consensus-based approach and decides the top-performing caller for each bin (Supplementary Tables 1–4). Next, VISTA merges the outputs of each of the top-performing callers into one output VCF file. This approach ensures that the best caller per organism, variant type, and variant length are selected, and the combination of several top callers exceeds existing callers. For VISTA’s discovery mode, where the ground truth is not known, we use the combination of callers identified during VISTA’s pre-training, on the organism closest to the input sample. VISTA’s input consists of four VCF files outputted by top-performing callers from each bin. Octopus [18], Manta [26], DELLY [28], and GenomeSTRiP [32] combinations were used for humans. LUMPY [40], Manta [26], Clever [27], and PopDel [29] combinations were used for mice. We have found VISTA’s pre-trained algorithm to be generalizable across multiple samples of an organism. If the user wants to compare the results of VISTA with other callers and chooses to provide a gold standard file, VISTA performs downstream analysis to produce statistics such as the sensitivity, precision, and F1-score, as well as graphs comparing the output of VISTA to other variant callers. In case a gold standard is lacking, the closest gold standard match to the input file is chosen, and the statistical analysis is performed using that gold standard.

### Comparing deletion inferred from whole-genome sequencing data with the gold standard

In order to compute the performance of the SV callers, we compared the deletions inferred by the SV callers from the WGS data, called inferred deletions with our molecular-based gold standard, called true deletions. The start and end positions of the deletion were taken into account when comparing true deletions with inferred deletions. The inferred deletion was considered to be correctly predicted if the distance between the left and right coordinates fell within the resolution threshold τ from the true deletion’s coordinates. We used a dynamic overlap threshold ranging from 10–10 000 bp, rather than a fixed percentage. The resolution threshold τ is set at values of 0, 10, 100, 1000, and 10 000 bp. Due to most tools yielding no matches at a 0 bp threshold, we set the initial threshold in the figures to 10 bp. We defined true positives as correctly predicted deletions that were defined as deletions reported by the SV caller and were also present in the gold standard. When an inferred deletion corresponds to multiple true deletions, we arbitrarily select one for matching.

Similarly, when a true deletion matches multiple inferred deletions, we select the first matching deletion. Deletions predicted by the SV caller but absent from the gold standard were classified as FP. We made sure to only match each deletion present in the gold standard with only one deletion predicted by the software. We defined false negatives (FN) as variants that were present in the gold standard but were not predicted by the SV caller. The accuracy of SV detection was evaluated across different detection thresholds (τ). The accuracy at threshold τ is defined as the percentage of SVs whose absolute error in deletion coordinates is less than or equal to τ. To evaluate the accuracy of SV callers, we have employed the following measures:

- Sensitivity = True Positive/ (True Positive +False Negative)
- Precision = True Positive/ (True Positive +False Positive)
- F-score = 2 * Sensitivity * Precision/ (Sensitivity + Precision)

### Downsample the whole-genome sequencing samples

To obtain the desired coverage, we have made a custom script to downsample the full coverage BAM file. Existing tools, such as samtools, are inappropriate for this purpose because they handle each read from a read pair independently, leading to singleton reads in the downsampled BAM file.

### Variant Identification and Structural Variant Analysis train-test experiments

For deletions, we trained VISTA on five different mouse strains on chr19 (A_J, AKR_J, BALB_CJ, CBA_J, C3H_HeJ) to find the best individual callers per length bin, and tested on DBA_2J and LP_J. For human data, we trained VISTA on chr 17, chr 18, and chr 19, and tested on chr 15 and chr16. We found Octopus, Manta, Delly, and GenomeStrip to be the optimal combination for HG002; Octopus, Manta, Delly, and Manta for HPRC; and Lumpy, Manta, Clever, and PopDel for MM7.

### Data availability

A_J, AKR_J, BALB_cJ, CBA_J, C3H_HeJ, DBA_2J, and LP_J samples from WGS mouse strains are all publicly available under the following accession numbers in the European Nucleotide Archive, including ERP000037, ERP000038, ERP000039, ERP000040, ERP000044, and ERP000045. Output VCFs from SV tools, gold standard VCF, figure images, and scripts used for analysis can be found at https://github.com/Mangul-Lab-USC/benchmarking_SV. The human high confidence bed file is accessible here https://www.nist.gov/programs-projects/genome-bottle.

The novoaligned BAMS data for the HG002_NA24385 son genome were accessible from https://ftp-trace.ncbi.nlm.nih.gov/ReferenceSamples/giab/data/AshkenazimTrio/HG002_NA24385_son/NIST_Illumina_2x250bps/novoalign_bams/.

The 10 human sample (HG00733, HG00438, HG00621, HG00735, HG00741, HG01071, HG01106, HG01109, HG01243, and HG01175) BAMS data are from the 1000 Genomes Project with 30× coverage on the reference GRCh38 [42]. They can be downloaded through the IGSR [43] portal at https://www.internationalgenome.org/data-portal/sample.

### Code availability

All code required to produce the figures and analysis performed in this paper is freely available at https://github.com/Mangul-Lab-USC/VISTA_paper.

### Tool availability

The source code and usage instructions for VISTA are open source under the MIT license. MIT license is a permissive software license recognized globally. The source is available at: https://github.com/Mangul-Lab-USC/VISTA.

## Results

### Variant Identification and Structural Variant Analysis: an integrated framework for structural variant discovery

VISTA is a novel consensus-based integrated framework for structural variant discovery, designed to overcome the challenges associated with variant calling. Leveraging multiple variant callers based on organism characteristics, genomic coverage, and variant length, VISTA dynamically adapts to diverse genomic contexts, ensuring precise and comprehensive SV detection. VISTA’s unique merging procedure combines the outputs of individual callers using a novel filtering and merging algorithm, resulting in a highly accurate SV set with minimized FP and FN. Additionally, VISTA offers an optimized mode for prioritizing either precision or recall, further enhancing its flexibility and applicability to diverse research objectives.

**Figure 2:**
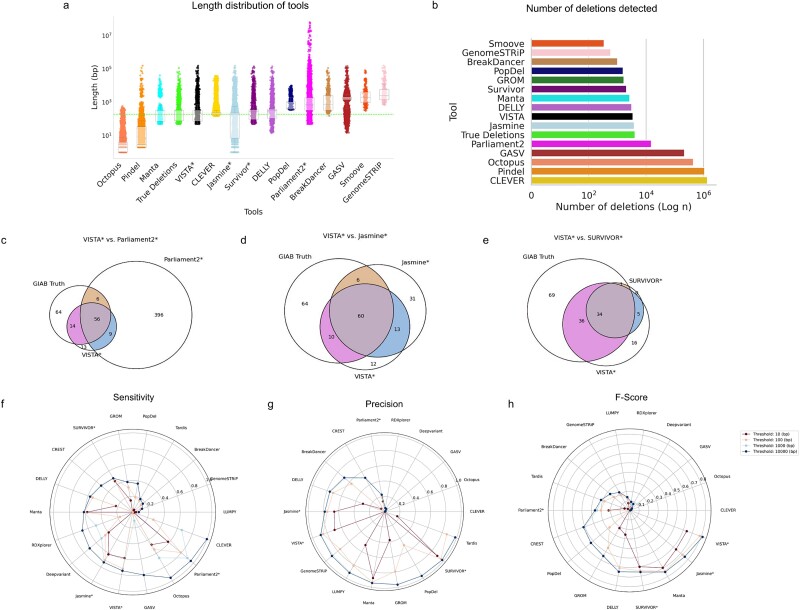
Evaluating the performance of VISTA against popular SV callers on GIAB-HC-D. A deletion is regarded as accurately predicted if the distance between the left and right coordinates is less than the resolution threshold of the true deletion coordinates. (a) Length distribution of VISTA versus GIAB-HC-D and other callers (b) Number of deletions detected by VISTA versus GIAB-HC-D and other callers. (c–e) Venn diagrams of intersecting top SV call sets on HG002 chr 19. (f) Sensitivity of SV callers at different thresholds. (g) Precision of SV callers at different thresholds. (h) F-score of SV callers at different thresholds. The asterisk represents consensus-based callers.

### Datasets used for this study

To assess the accuracy of VISTA, we used datasets obtained from various organisms, read lengths, and genomic coverages. We used 17 WGS samples from three major datasets, referred to as D1, D2, and D3. These datasets were used to compare the performance of VISTA with 18 individual SV callers and three consensus-based callers, using comprehensive gold standards, namely GIAB (GIAB-HC), HPRC (HPRC-HC), and *Mus musculus* (MM-7). D1 consists of the HG002 Ashkenazi Jewish Trio son sample from the GIAB consortium [30]. The alignment files were publicly available from the GIAB website and were used as input to the SV callers. The average depth of coverage was 45× and the reads were 2 × 250 bp. For GIAB-HC, we use the GIAB preliminary variant set containing 37 412 deletions in HG002. We then extracted the high-confidence regions using the high-confidence bed file and variants greater than 50 bp, since events less than 50 bp are too small to lie within the definition of structural variants [41]. This leads us to obtain our gold standard set of deletions consisting of 4120 deletions (GIAB-HC-D). For insertions, we used the GIAB preliminary variant set consisting of 35 163 insertions (GIAB-I). D2 refers to 10 WGS human samples from the 1000 Genomes Project. The average depth of coverage was 30× and the reads were 2 × 150 bp. For the HPRC-HC dataset, we used haplotype-resolved de novo assemblies from the HPRC to generate benchmarking SV sets. We use the high-quality HPRC assemblies as the ground truth, and use dipcall to extract the structural variations from the assemblies. Because dipcall can find structural variations from assemblies with high-confidence, we use dipcall with the high-quality HPRC assemblies as the truth for benchmarking. On average, each set contains 17 807 deletions larger than 50 bp (Supplementary Fig. 3c). Dataset D3 refers to seven inbred mouse WGS samples. The high-coverage sequence data was used as an input to the SV callers in the form of aligned reads for all experiments. For 7MM-HC [13], we used a PCR-validated set of deletions, in which the mouse deletions were manually curated. To precisely determine the ends of each deletion at the base pair, we employed targeted PCR amplification of the breakpoints followed by sequencing (Supplementary Table 2).

### Evaluation metrics

Callers were evaluated based on their ability to detect structural variants using precision, sensitivity, and F-score as metrics. These metrics were computed using a system of resolution thresholds that varied from 10–10 000 bp. An inferred deletion was regarded as accurately predicted if the distance between the left and right coordinates was less than the resolution threshold of the true deletion coordinates.

### Variant Identification and Structural Variant Analysis achieves the optimal balance of precision and sensitivity for deletion calls across two independent human benchmarks

We assessed the performance of VISTA in terms of precision (false discovery rate), recall (true positive rate), compared to other individuals (e.g. DELLY [28], Manta [26], Lumpy [40]), and consensus-based (e.g. Parliament2 [19], SURVIVOR [21], and Jasmine [20]) short-read SV methods across 11 WGS samples from D1 and D2 datasets with gold standard sets available as a part of GIB-HC-D and HPRC-HC.

First, we compared the length distribution of the deletions in the GIB-HC, compared to VISTA and other callers. VISTA and Manta [26] were the top 2 callers with a median length closest to the gold standard (Fig. 2a). Notably, all of the consensus-based variant callers overestimate the deletion length by 175 bp on average. Parliament2 [19] was the only consensus caller that overestimated the number of deletions, while all other callers underestimated. We plotted Venn diagrams to better visualize overlapping deletion call sets for VISTA, the gold standard, and other top-performing variant callers for HG002 chr19 (Fig. 2c–e). Callers either had too many FP (Parliament2 [19], Jasmine [20]), detected too few deletions (SURVIVOR [21]), or had a smaller overlap with the ground truth (Manta [26]). We observed a high concordance of deletions predicted by VISTA and the ground truth set. Next, we compared the performance of VISTA with other SV callers in terms of inferring deletions for a range of resolution thresholds from 10–10 000 bp (Fig. 2f–h). VISTA has achieved the highest recall at 100 bp (72.4%) while having the fourth-highest precision (85.7%). Only SURVIVOR [21] (95.9%), Manta [26] (93.4%), and Tardis [36] (86.3%) had a higher precision, at the cost of a lower recall: SURVIVOR [21] (48.3%), Manta [26] (61.0%), Tardis [36] (16.8%). Importantly, VISTA achieves the highest F1 score (78.5%) for thresholds 100 bp and above, followed by Manta [26] (73.8%) and Jasmine [20] (72.4%; Fig. 2h). For a threshold of 10 bp, Manta [26] (69.5%) had a marginally higher F-score than VISTA (63.8%), followed by Jasmine [20] (63.6%).

**Figure 3:**
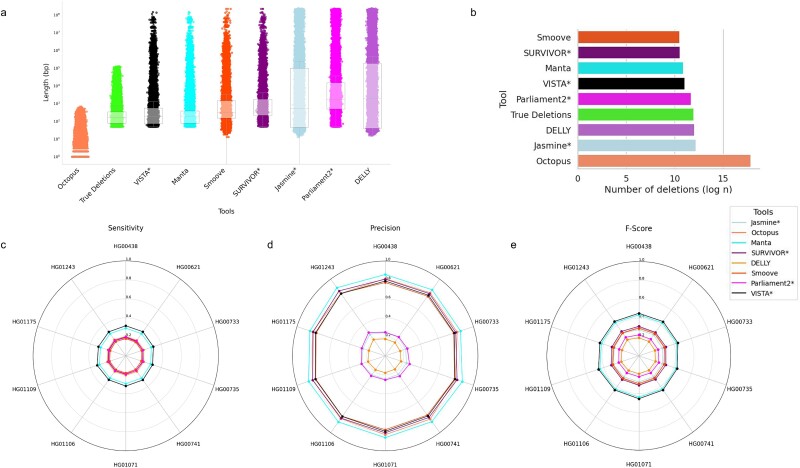
Evaluating the performance of VISTA against popular SV callers on HPRC-HC. A deletion is regarded as accurately predicted if the distance between the left and right coordinates is less than the resolution threshold of the true deletion coordinates. (a) Length distribution of VISTA and other top callers. Figures are ordered in increasing median deletion length (b) number of deletions detected by VISTA versus HPRC-HC and other callers (c) sensitivity of VISTA versus other callers for 10 samples (d) precision of VISTA versus other callers for 10 samples (e) F-score of VISTA versus other callers for 10 samples. The asterisk denotes consensus-based callers.

Next, we studied the robustness of VISTA across 10 human samples on the HPRC dataset (Fig. 3). VISTA had the closest median deletion length compared to the gold standard (Fig. 3a). All of the consensus-based callers underestimated the number of deletions (Fig. 3b). We observed all the callers to have a consistent trend across the samples, with VISTA consistently achieving the highest F-score across all samples (Fig. 3e). Most samples demonstrated a slightly elevated precision for samples HG00735 and HG01234, and a minimum at sample HG01175 (Fig. 3d).

### Variant Identification and Structural Variant Analysis maintains high performance for deletion calls across *Mus musculus* samples

**Figure 4:**
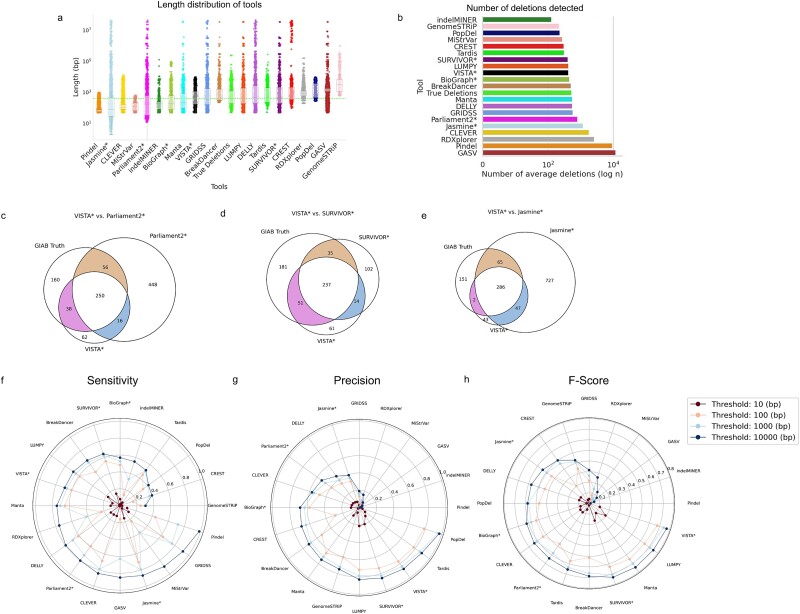
Evaluating the performance of VISTA against popular SV callers on mouse data. A deletion is regarded as accurately predicted if the distance between the left and right coordinates is less than the resolution threshold of the true deletion coordinates (a) Length distribution of VISTA versus mouse gold standard versus other callers. Figures are ordered in increasing median deletion length (b) Number of deletions detected by VISTA versus mouse gold standard versus other callers (c–e) Venn diagrams of GIAB truth and VISTA with other top callers on AKR_J mouse strain. The asterisk represents consensus-based callers. (f) Sensitivity of SV callers at various thresholds. (g) Precision of SV callers at various thresholds. (h) F-score of SV callers at various thresholds. The results displayed above report the average across seven different mouse strains.

We compared the performance of VISTA with 16 individual SV callers and 3 consensus-based SV callers in terms of inferring deletions on 7MM-HC. We compared the deletion lengths predicted by VISTA and other consensus callers to the ground truth. VISTA was the closest in terms of the length distribution of deletions as compared to 7MM-HC (Fig. 4a). We plotted Venn diagrams to better visualize overlapping deletion call sets for VISTA, the gold standard, and other top-performing variant callers for 7MM chr19 (Fig. 4c–e). Similar to human data, callers either had too many FP (Parliament2 [19] and Jasmine [20]), or detected too few deletions (SURVIVOR [21]). We observed a high concordance of deletions predicted by VISTA and the ground truth set. We analyzed the performance to detect mouse deletions for a range of resolution thresholds from 10 to 10 000 bp (Fig. 4 f–h). VISTA has the third highest precision (77%) at 100 bp and above (Fig. 4g). Only TARDIS [36] (78%) and PopDel [29] (79%) are marginally higher, at the cost of a significantly lower recall. Several tools such as Jasmine [20] (70%) and GRIDSS [35] (63%) outperform VISTA (61%) in terms of Sensitivity but are among the lowest-performing tools for precision (Fig. 4f). Thus, VISTA (68%) has the highest F1 score for thresholds 100 bp and above, followed by Manta [26] (65.0%) and LUMPY [40] (64.8%; Fig. 4h). For a threshold of 10 bp, LUMPY [40] (19%), SURVIVOR [21] (17%), and Manta [26] (11.2%) have a marginally higher F-score than VISTA (10.6%). Among the consensus-based callers, SURVIVOR [21] achieves the highest F-score (63%) at 100 bp and above, followed by Parliament2 [19] (48%) and Jasmine [20] (44%). Results across the individual mouse strains are depicted in Supplementary Fig. 1.

### Variant Identification and Structural Variant Analysis delivers highly accurate insertion calls

**Figure 5:**
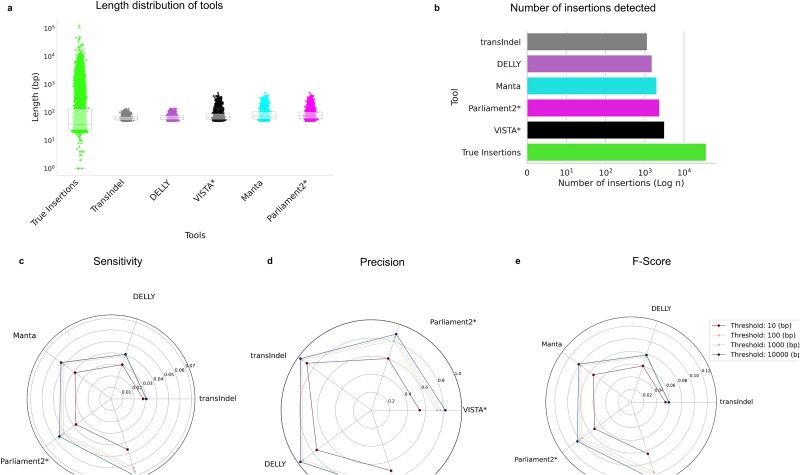
Evaluating the performance of VISTA against popular SV callers on GIAB-HC-I. An insertion is regarded as accurately predicted if the distance between the left and right coordinates is less than the resolution threshold of the true deletion coordinates. (a) Length distribution of VISTA versus GIAB-HC-I and other callers (b) Number of insertions detected by VISTA versus GIAB-HC-I and other callers (c) Sensitivity of SV callers at various thresholds (d) Precision of SV callers at various thresholds (e) F-score of SV callers at various thresholds. The asterisk represents consensus-based callers.

We compared the performance of VISTA with several other consensus-based callers in terms of inferring insertions on GIAB-I. In contrast to deletions, the length distribution of detected insertions varied across tools, and differed significantly from the true distribution (Fig. 5a). Notably, none of the callers demonstrated the capability to accurately predict long insertions (>1000 bp). Hence, all the callers underestimated the number of insertions (Fig. 5b). We found VISTA and Parliament2 [19] to be the two callers with median insertion lengths of 16 and 66 bp closest to the gold standard (44 bp; Fig. 5a). To assess the performance of VISTA against other callers in detecting insertions, we analyzed a range of resolution thresholds spanning from 10–10 000 bp (Fig. 5c–e). VISTA has the highest recall at 100 bp (6.3%) while having the fifth-highest precision (65%) at 100 bp. While both transIndel [39] (95.40%) and Manta [26] (94.31%) had a higher precision, this came at the expense of a lower recall. Thus, VISTA (11.6%) had the highest F1 score for thresholds 10 bp and above, followed by Parliament2 [19] and Manta [26] (9.8%) at 100 bp.

### Variant Identification and Structural Variant Analysis achieves the highest performance on low-coverage whole-genome sequencing samples

**Figure 6:**
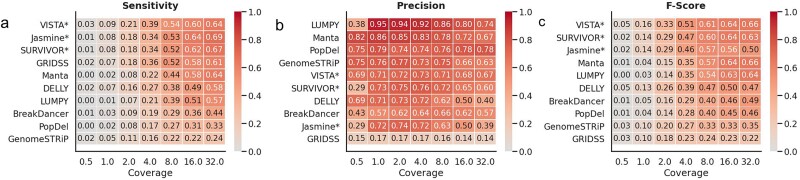
Evaluating the performance of VISTA against popular SV callers on low-coverage mouse data. (a) Heatmap depicting the sensitivity based on 100 bp threshold, shown across varying coverage depths. (b) Heatmap depicting the precision based on 100 bp threshold, shown across varying coverage depths. (c) Heatmap depicting the F-score based on 100 bp threshold, shown across varying coverage depths. The asterisk represents consensus-based callers.

We evaluated the VISTA’s performance, alongside other SV callers, using original mouse WGS data downsampled at various coverage levels (Fig. 6). Ten subsamples were generated for each coverage level varying from 0.1×–32×. In general, for each method, the number of correctly detected deletions declined as the coverage depth declined. VISTA was able to obtain the highest F-score consistently across all coverages from 0.5×–32×, closely followed by Delly for 0.5×, SURVIVOR [21], and Jasmine [20] for 1×–8×. For higher coverages ranging from 16×–32×, SURVIVOR [21] Manta [26], and LUMPY [40] were the top 3 tools closest in performance to VISTA (Fig. 6c). VISTA obtained a maximum precision of 0.73 at an intermediate coverage of 4× (Fig. 6b). While methods like LUMPY [40] were able to achieve a higher precision of up to 0.94, this came at the cost of a lower sensitivity. As the coverage increased, the sensitivity and F-score increased, with a maximum value of 0.64 and 0.66 respectively, at 32× coverage (Fig. 6a).

### Variant Identification and Structural Variant Analysis offers an additional mode to boost previsions or sensitivity

**Figure 7:**
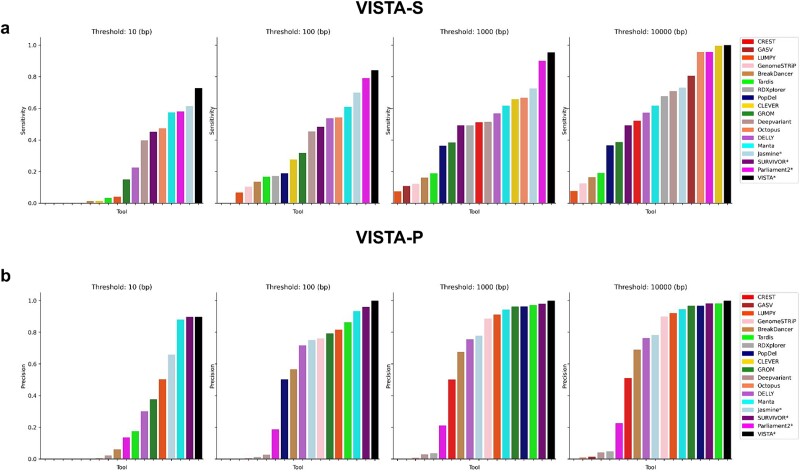
Evaluating the performance of VISTA-optimized against popular SV callers on human data. A deletion is regarded as accurately predicted if the distance between the left and right coordinates is less than the resolution threshold of the true deletion coordinates. (a) Sensitivity of VISTA-S versus other SV callers at various thresholds. (b) Precision of VISTA-P versus other SV callers at different thresholds.

We recognize that certain applications may require a highly precise variant caller or a caller with a high recall. Hence, we have provided an ‘optimize mode’ in VISTA, where the user can choose to optimize for either precision or recall. We compared the sensitivity of VISTA-optimized as compared to other SV callers (Fig. 7a). VISTA (85%) obtains the highest sensitivity consistently across all thresholds from 10–10 000 bp and is significantly higher than the second and third top-performing callers, Jasmine [20] (70%) and Manta [26] (65%). We also compared the precision of VISTA compared to other SV callers (Fig. 7b), and VISTA (100%) obtains the highest precision at 100 bp and above with no FP, closely followed by SURVIVOR [21] (95%) and Manta [26] (93%).

### Computational performance of Variant Identification and Structural Variant Analysis

We compared the central processing unit (CPU) and random access memory (RAM) requirements of VISTA with other popular consensus-based callers (more details in Supplementary materials, Supplementary Table 9). VISTA and SURVIVOR [21] were the two callers with the lowest RAM usage, with 7 MB of RAM used to process the human sample from the GIAB-HC-D dataset. Additionally, VISTA has a significantly low CPU time, of 0.65 seconds, as compared to Parliament2 [19] (2.3 hours) and Jasmine [20] (11.5 seconds). While certain consensus callers such as VISTA, Jasmine, and SURVIVOR take individual callers and input and only run the consensus algorithm, callers like Parliament2 run the individual callers through a Docker-based image, resulting in a higher run time. In order to perform a fair run-time comparison for the callers, we excluded the run time of the individual callers for Parliament2. We observed VISTA to be highly scalable across 10 samples, data of varying coverage.

## Discussion

Here, we report the development and evaluation of VISTA, an integrated structural variant calling framework, that leverages the results of individual callers using a novel and robust filtering and merging algorithm. Our study is the first to comprehensively benchmark the performance of a novel SV detection method against the HPRC benchmark. HPRC samples were selected to cover more genetic diversity than using the solo HG002 genome, in order to assess our method on a comprehensive and broad scale. We demonstrate VISTA’s abilities in being the only SV caller that can obtain a consistently high performance across variant types, datasets, coverages, and organisms.

While the recall ability for insertions is reduced due to limitations of short-read–based insertion detection algorithms, VISTA was able to obtain the highest F-score among other popular individual and consensus-based callers. We attempted to additionally train the insertion call set on SVseq2 and inGAP-sv [45], but these tools failed to detect both the start and end positions of insertions. All the SV callers we used for training had limitations on finding large insertions, where the largest insertion size Manta [26] outputs predicted is 503 bp, and the gold standard has 3568 insertions that exceed 500 bp across the full chromosome. Ultimately, VISTA merges Manta [26] and transIndel [39] to maximize the F-score and outperform other callers.

We observed Parliament2’s output to be highly inconsistent across human samples. For certain samples, it was only able to run a subset of the callers (Supplementary Table 6). This behavior is consistent with what other researchers faced (https://github.com/dnanexus-archive/parliament2/issues/62). DELLY [28], CNVnator [46], and LUMPY [40] were the only 3 callers that Parliament2 ran across all 10 human samples. To provide a fair comparison across the samples, we only considered the outputs of the three callers. While we used a custom script to compare the performance of the callers with the gold standard, we reran our analysis using Truvari [47], a tool extensively used by the community for assessing SV calls, and found our results to be consistent. In this study, the output of the SV callers was not filtered. Filtering is highly context-dependent and can differ greatly between experiments. Effective post-hoc filtering involves carefully evaluating the quality metric and thresholds of each tool to ensure consistent results.

We have performed additional experiments to ensure the generalizability of VISTA. In addition to VISTA’s superior performance when applied to datasets it was pre-trained on, VISTA is able to perform well on new datasets not included in the training data. For instance, when VISTA was trained on the HPRC dataset, it achieved the highest performance when evaluated on 10 different human HPRC samples with comparable characteristics (Fig. 3). Furthermore, when VISTA pre-trained on the HPRC dataset was applied to the HG002 (GIAB) sample, which it was not pre-trained on, the results remained the highest performing (Supplementary Fig. 4). This suggests that VISTA’s results are not due to overfitting, and the algorithm can generalize well across novel datasets with significantly different characteristics.

**Key Points**

- Previous studies have shown that none of the existing SV callers can maintain high accuracy across various SV lengths and genomic coverages
- VISTA overcomes this limitation by executing various combinations of top-performing callers based on variant length and genomic coverage to generate SV events with high accuracy
- VISTA represents a significant advancement in structural variant calling, offering a robust and accurate framework that outperforms existing consensus-based tools and sets a new standard for SV detection in genomic research

## Supplementary Material

Supplementary_bbae462_bbae462
