# Supplementary material for: VISTA: an integrated framework for structural variant discovery
Source: Brief Bioinform. 2024 Sep 19;25(5):bbae462. doi: 10.1093/bib/bbae462 (PMC11411772; doi:10.1093/bib/bbae462)
Supplement: Supplementary_bbae462_bbae462 [file supplementary_bbae462_bbae462.zip › Supplementary_bbae462/Supplementary Materials.docx]

**Supplementary Materials:**

**Compare the computational performance of SV callers**

The CPU time and RAM of each tool were measured to determine its computational performance. The statistics were measured for 1x coverage and full coverage bam files, with samples A/J and BALB/cJ for mouse data. The CPU time was computed using either the GNU time program that is built into make bash terminals or the Hoffman2 Cluster qsub command. For GNU time, we used this specific command /usr/bin/time -f "%e\t%U\t%S\t%M" which we either had to run manually on an interactive qsub session or through another method that wasn’t a qsub. This GNU time command would output one line containing Wallclock time in seconds, user time in seconds, kernel-space time in seconds, and peak memory consumption of the process in kilobytes. CPU time was calculated by adding user time and kernel space time. RAM usage was equivalent to peak memory consumption in the case of this command. For qsubs on the Hoffman2 Cluster, we used the command qsub -m e which would email the user a full list of records when the tool finished running. This list included CPU time and Max mem which was designated as RAM usage for each tool.
