# Supplementary material for: VISTA: an integrated framework for structural variant discovery
Source: Brief Bioinform. 2024 Sep 19;25(5):bbae462. doi: 10.1093/bib/bbae462 (PMC11411772; doi:10.1093/bib/bbae462)
Supplement: Supplementary_bbae462_bbae462 [file supplementary_bbae462_bbae462.zip › Supplementary_bbae462/Supplementary_Table_1.docx]

| Strain | 50-100 | 100-500 | 500-1000 | 1000+ | Results [VISTA, |
| --- | --- | --- | --- | --- | --- |
|  |  |  |  |  | Parliament2, |
|  |  |  |  |  | Survivor] |
|  |  |  |  |  |  |
| CHR17 | Octopus | Manta | Delly | Delly | [0.77,0.21,0.53] |
| CHR18 | Octopus | Manta | Delly | GENOMESTRIP | [0.89,0.45,0.71] |
| CHR19 | Octopus | Manta | Delly | GENOMESTRIP | [0.71,0.21,0.46] |
| **CHR15** | **Octopus** | **Manta** | **Delly** | **GENOMESTRIP** | **[0.75,0.44,0.71]** |
| **CHR16** | **Octopus** | **Manta** | **Delly** | **GENOMESTRIP** | **[0.73,0.23,0.67]** |
|  |  |  |  |  |  |

**Table S1:** Train-test distribution for human HG002 WGS data. VISTA was trained on chromosomes 17-19 to determine the highest performing caller per length bin and was tested on chromosomes 15 and 16.
