# Supplementary material for: VISTA: an integrated framework for structural variant discovery
Source: Brief Bioinform. 2024 Sep 19;25(5):bbae462. doi: 10.1093/bib/bbae462 (PMC11411772; doi:10.1093/bib/bbae462)
Supplement: Supplementary_bbae462_bbae462 [file supplementary_bbae462_bbae462.zip › Supplementary_bbae462/Supplementary_Table_2.docx]

| Sample | 50-100 | 100-500 | 500-1000 | 1000+ |
| --- | --- | --- | --- | --- |
|  |  |  |  |  |
| A_J | Lumpy | Manta | Manta | Manta |
|  |  |  |  | /Popdel |
| AKR_J | Lumpy | Manta/GRIDSS | MANTA | POPDEL |
| BALB_CJ | Clever | MANTA | CLEVER | MANTA |
| C3H_HeJ | Lumpy | MANTA | POPDEL | CLEVER/ |
|  |  |  |  | POPDEL |
| CBA_J | LUMPY | MANTA | CLEVER | MANTA |
| **DBA_2J** | **LUMPY** | **MANTA** | **CLEVER** | **POPDEL** |
| **LP_J** | **LUMPY** | **MANTA** | **CLEVER** | **POPDEL** |

**Table S2**: Train-test distribution for 7MM. VISTA was trained on 6 different mouse strains to determine the highest performing caller per bins and was tested on chromosomes DBA_2J and LP_J.
