# Supplementary material for: VISTA: an integrated framework for structural variant discovery
Source: Brief Bioinform. 2024 Sep 19;25(5):bbae462. doi: 10.1093/bib/bbae462 (PMC11411772; doi:10.1093/bib/bbae462)
Supplement: Supplementary_bbae462_bbae462 [file supplementary_bbae462_bbae462.zip › Supplementary_bbae462/Supplementary_Table_3.docx]

| Length Bin | | 0.5x | 1.0x | 2.0x | 4.0x | 8.0x | 16.0x | 32.0x |
| --- | --- | --- | --- | --- | --- | --- | --- | --- |
|  |  |  |  |  |  |  |  |  |
| 50 - 100 |  | N/A | N/A | GRIDSS | GRIDSS | GRIDSS | MANTA | MANTA |
| 100 - 500 |  | GRIDSS | GRIDSS | GRIDSS | GRIDSS | GRIDSS | GRIDSS | MANTA |
| 500 | - | DELLY/GRI | DELLY | DELLY | DELLY | DELLY | DELLY/BRE | BREAKDA |
| 1000 |  | DSS |  |  |  |  | AKDANCE | NCER/DEL |
|  |  |  |  |  |  |  | R | LY/MANTA |
| 1000 + |  | GENOMES | DELLY | DELLY | DELLY | DELLY | DELLY/LU | DELLY/GE |
|  |  | TRIP/DELL |  |  |  |  | MPY/MAN | NOMESTR |
|  |  | Y |  |  |  |  | TA | IP/MANTA |
|  |  |  |  |  |  |  |  |  |

**Table S3**: Training based on the f-score for downsampled data
