# Supplementary material for: VISTA: an integrated framework for structural variant discovery
Source: Brief Bioinform. 2024 Sep 19;25(5):bbae462. doi: 10.1093/bib/bbae462 (PMC11411772; doi:10.1093/bib/bbae462)
Supplement: Supplementary_bbae462_bbae462 [file supplementary_bbae462_bbae462.zip › Supplementary_bbae462/Supplementary_Table_4.docx]

| Sample | 50-100 | 100-500 | 500-1000 | 1000+ |
| --- | --- | --- | --- | --- |
|  |  |  |  |  |
| HG00438 | Octopus | Manta | DELLY | Manta/Smoove |
| HG00621 | Octopus | Manta | DELLY | Manta/Smoove |
| HG00733 | Octopus | Manta | DELLY | Manta/Smoove |
|  |  |  |  |  |

**Table S4**: Train-test distribution for HPRC-HC. VISTA was trained on HG00438, HG00621, HG00733 to

determine the highest performing caller per length bin.
