# Supplementary material for: VISTA: an integrated framework for structural variant discovery
Source: Brief Bioinform. 2024 Sep 19;25(5):bbae462. doi: 10.1093/bib/bbae462 (PMC11411772; doi:10.1093/bib/bbae462)
Supplement: Supplementary_bbae462_bbae462 [file supplementary_bbae462_bbae462.zip › Supplementary_bbae462/Supplementary_Table_5.docx]

| **Strain name** | **Total number** | **Average** | **Minimum** | **Maximum** |
| --- | --- | --- | --- | --- |
|  | **of gold** | **deletion length** | **deletion length** | **deletion length** |
|  | **standard** |  |  |  |
|  | **deletions** |  |  |  |
|  |  |  |  |  |
| A/J | 533 | 1968 | 15 | 239572 |
| AKR/J | 504 | 1400 | 13 | 24301 |
| BALB/cJ | 545 | 1947 | 15 | 239572 |
| C3H/HeJ | 539 | 1993 | 15 | 239572 |
| DBA/2J | 609 | 1301 | 16 | 24301 |
| LP/J | 483 | 1534 | 13 | 24301 |
| CBA/J | 586 | 1330 | 24 | 24301 |
|  |  |  |  |  |

**Table S5:** Gold standard deletion calls from chromosome 19 from 7 inbred mouse strains.
