# Supplementary material for: VISTA: an integrated framework for structural variant discovery
Source: Brief Bioinform. 2024 Sep 19;25(5):bbae462. doi: 10.1093/bib/bbae462 (PMC11411772; doi:10.1093/bib/bbae462)
Supplement: Supplementary_bbae462_bbae462 [file supplementary_bbae462_bbae462.zip › Supplementary_bbae462/Supplementary_Table_6.docx]

| Samples | Breakdancer | CNVnator | Delly2 | Lumpy | Manta | BreakSeq2 |
| --- | --- | --- | --- | --- | --- | --- |
|  |  |  |  |  |  |  |
| HG00733 | Yes | Yes | Yes | Yes | No | No |
| HG00438 | Yes | Yes | Yes | Yes | No | No |
| HG00621 | Yes | Yes | Yes | Yes | Yes | No |
| HG00735 | No | Yes | Yes | Yes | Yes | Yes |
| HG00741 | No | Yes | Yes | Yes | Yes | Yes |
| HG01071 | Yes | Yes | Yes | Yes | No | No |
| HG01106 | Yes | Yes | Yes | Yes | No | No |
| HG01109 | Yes | Yes | Yes | Yes | Yes | No |
| HG01243 | No | Yes | Yes | Yes | Yes | Yes |
| HG01175 | No | Yes | Yes | Yes | Yes | Yes |
| HG002 | No | Yes | Yes | Yes | Yes | No |

**Table S6**: Parliament2 results on different human samples. Parliament2 outputs results including different callers.
