# Supplementary material for: VISTA: an integrated framework for structural variant discovery
Source: Brief Bioinform. 2024 Sep 19;25(5):bbae462. doi: 10.1093/bib/bbae462 (PMC11411772; doi:10.1093/bib/bbae462)
Supplement: Supplementary_bbae462_bbae462 [file supplementary_bbae462_bbae462.zip › Supplementary_bbae462/Supplementary_Table_7.docx]

| **Tool** | **Parameter Args** | **Parameter Option** |
| --- | --- | --- |
|  |  |  |
| Pindel | None | None |
| Popdel | -d | Maximum SV size of deletions |
| Manta | None | None |
| DELLY | None | None |
| CLEVER | None | None |
| BREAKDANCER | -m INT | Maximum SV size |
|  |  | But default: 1000000000 |
| CREST | None | None |
| LUMPY | None | None |
| Smoove | None | None |
| Tardis | None | None |
| RDXPlorer | None | None |
| GROM | None | None: |
| Octopus | None | None |
| GRIDSS | None | None |
| VISTA | None | None |
|  |  |  |

**Table S7:** SV lengths parameter settings for different callers
