# Supplementary material for: VISTA: an integrated framework for structural variant discovery
Source: Brief Bioinform. 2024 Sep 19;25(5):bbae462. doi: 10.1093/bib/bbae462 (PMC11411772; doi:10.1093/bib/bbae462)
Supplement: Supplementary_bbae462_bbae462 [file supplementary_bbae462_bbae462.zip › Supplementary_bbae462/Supplementary_Table_8.docx]

|  | 50-100 | 100-500 | 500-1000 | 1000+ | Final |
| --- | --- | --- | --- | --- | --- |
|  |  |  |  |  | F-Score |
|  |  |  |  |  |  |
| Default | Octopus | Manta | DELLY | GENOMESTRiP | 0.78 |
|  |  |  |  |  |  |

|  | 50-80 | 80-250 | 250-350 | 350+ | Final |
| --- | --- | --- | --- | --- | --- |
|  |  |  |  |  | F-Score |
|  |  |  |  |  |  |
| Case 1 | Octopus | Manta | Manta | Popdel | 0.64 |
|  |  |  |  |  |  |

|  | 50-80 | 80-250 | 250-350 | 350-1000 | 1000+ | Final |
| --- | --- | --- | --- | --- | --- | --- |
|  |  |  |  |  |  | F-Score |
|  |  |  |  |  |  |  |
| Case 2 | Octopus | Manta | Manta | Popdel | GENOMESTRiP | 0.78 |
|  |  |  |  |  |  |  |

**Table S8:** Different combinations of length bins and their f-score for VISTA on HG002 sample
