# Supplementary material for: VISTA: an integrated framework for structural variant discovery
Source: Brief Bioinform. 2024 Sep 19;25(5):bbae462. doi: 10.1093/bib/bbae462 (PMC11411772; doi:10.1093/bib/bbae462)
Supplement: Supplementary_bbae462_bbae462 [file supplementary_bbae462_bbae462.zip › Supplementary_bbae462/Supplementary_Table_9.docx]

|  | SURVIVOR | Parliament2 | Jasmine | VISTA |
| --- | --- | --- | --- | --- |
|  |  |  |  |  |
| CPU Time (s) | 0.10 | 8400 | 11.5 | 0.65 |
| RAM Usage (GB) | 0.00656 | 8.59 | 1.377 | 0.007004 |
|  |  |  |  |  |

**Table S9**: **A comparison of the computational performance of SV consensus-based callers**. The bar plot

depicts the CPU and RAM usage across all of the tools.
