# Supplementary figures and images for: VISTA: an integrated framework for structural variant discovery
Source: Brief Bioinform. 2024 Sep 19;25(5):bbae462. doi: 10.1093/bib/bbae462 (PMC11411772; doi:10.1093/bib/bbae462)

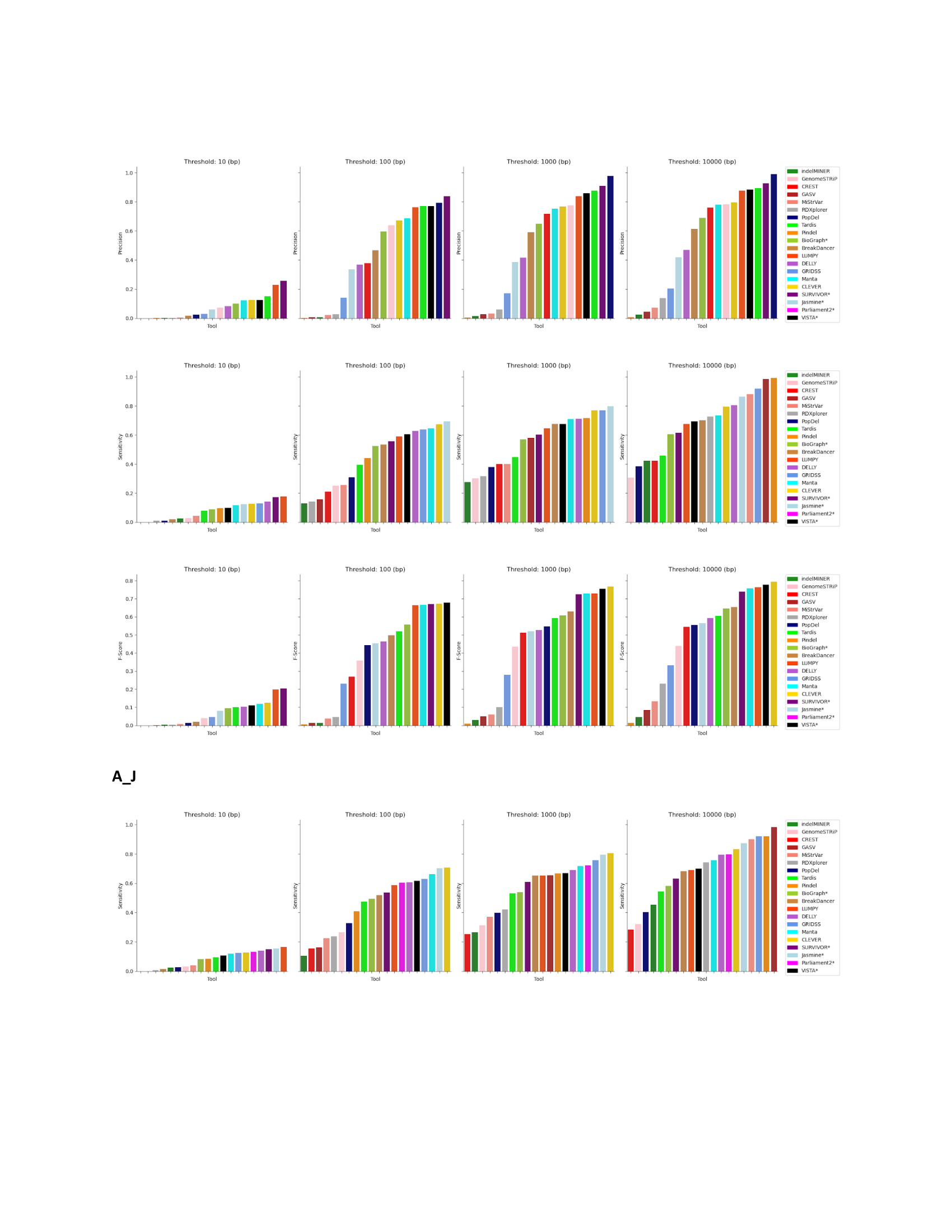

Supplement: Supplementary_bbae462_bbae462 [file supplementary_bbae462_bbae462.zip › Supplementary_bbae462/Supplementary Figure 1.tiff]

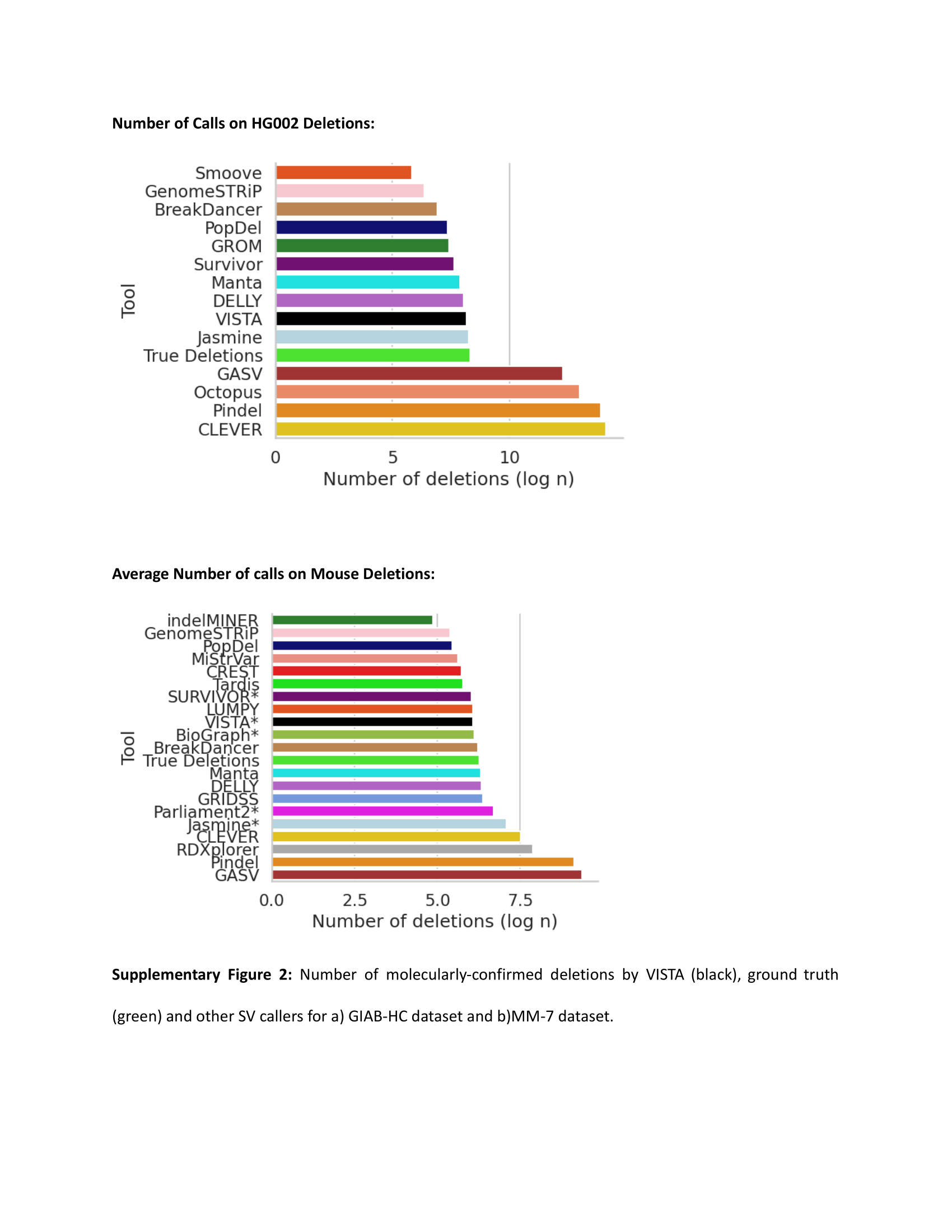

Supplement: Supplementary_bbae462_bbae462 [file supplementary_bbae462_bbae462.zip › Supplementary_bbae462/Supplementary_Figure_2.tiff]

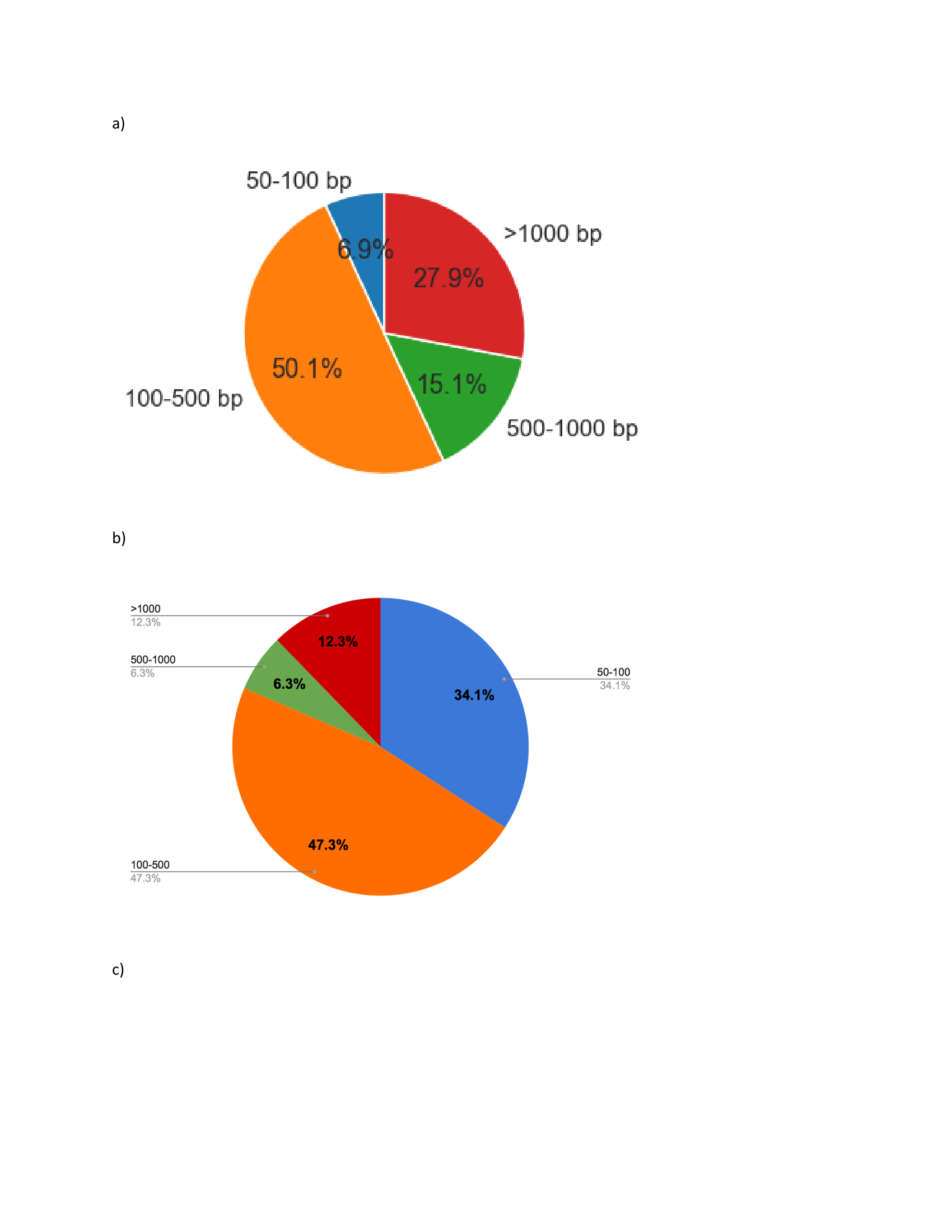

Supplement: Supplementary_bbae462_bbae462 [file supplementary_bbae462_bbae462.zip › Supplementary_bbae462/Supplementary_Figure_3.tiff]

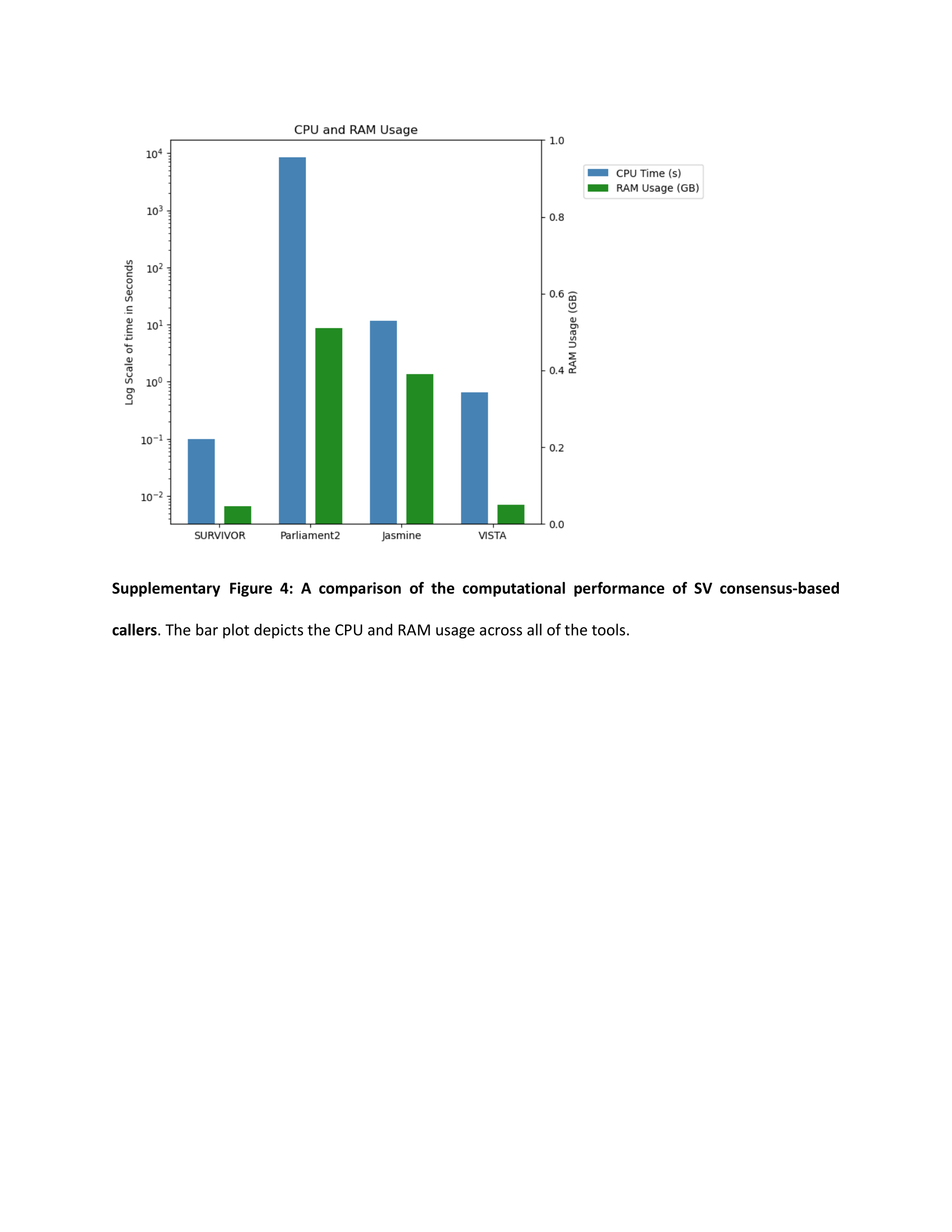

Supplement: Supplementary_bbae462_bbae462 [file supplementary_bbae462_bbae462.zip › Supplementary_bbae462/Supplementary_Figure_4.tiff]
